# Knowledge and utilization of intermittent preventive treatment for malaria among pregnant women attending antenatal clinics in primary health care centers in rural southwest, Nigeria: a cross-sectional study

**DOI:** 10.1186/1471-2393-9-28

**Published:** 2009-07-09

**Authors:** Stella O Akinleye, Catherine O Falade, Ikeoluwapo O Ajayi

**Affiliations:** 1 Department of Epidemiology, Medical Statistics and Environmental Health, College of Medicine, University of Ibadan, Nigeria; 2 Department of Pharmacology and Therapeutics, College of Medicine, University of Ibadan, Oyo State, Nigeria

## Abstract

**Background:**

Intermittent preventive treatment for prevention of malaria in pregnancy (IPTp) is a key component of malaria control strategy in Nigeria and sulfadoxine-pyrimethamine (SP) is the drug of choice. Despite the evidence of the effectiveness of IPTp strategy using SP in reducing the adverse effects of malaria during pregnancy the uptake and coverage in Nigeria is low. This study set out to assess the use of IPTp among pregnant women attending primary health centres in the rural area and determine factors that influence the uptake.

**Methods:**

A cross-sectional study was carried out between July and August 2007 among 209 pregnant women selected by systematic random sampling from antenatal care attendees at primary health care in a rural Local Government Area of Ekiti State, Nigeria. Information on knowledge of IPT, delivery, adherence and acceptability was obtained using an interviewer administered questionnaire. Descriptive statistics such as means, range, proportions were used. Chi-square test was used to examine association between categorical variables. All analyses were performed at 5% level of significance.

**Results:**

One hundred and nine of 209 (52.2%) respondents have heard about IPTp but only 26 (23.9%) were able to define it. Fifty seven (27.3%) reported to have received at least one dose of IPTp during the index pregnancy and all were among those who have heard of IPTp (52.3%). Twenty one of the 57 (36.8%) took the SP in the clinic. Only three of the twenty-one (14.3%) were supervised by a health worker. Twenty two of the 36 women (61.1%) who did not take their drugs in the clinic would have liked to do so if allowed to bring their own drinking cups. Almost half (43.9%) of those who had used IPTp during the index pregnancy expressed concern about possible adverse effect of SP on their pregnancies. Periodic shortages of SP in the clinics were also reported.

**Conclusion:**

In this study, IPTp use among pregnant women was very low and there was poor adherence to the Directly Observed Therapy (DOT) scheme. Concerted effort should be made to increase awareness of IPTp among the public especially women of child bearing age. Health workers should also be trained and monitored to ensure adherence.

## Background

Each year, more than 30 million African women in malaria endemic areas become pregnant and are at risk of infection with *Plasmodium falciparum *[1,2]. This results in high prevalence of patent parasitemia and clinical malaria [3,4] in pregnancy. In southwest Nigeria, past studies reported malaria parasite prevalence of between 60% and 72% among pregnant women [4,5]. Malaria during pregnancy causes up to 10,000 maternal deaths each year and contributes to high rates of maternal morbidity including fever and severe anemia, especially in first time mothers [6,7]. It is also a cause of low birth weight and placental parasitaemia [8,9]. Between 75,000 to 200,000 infant deaths annually are attributable to malaria infection in pregnancy [10,11]. A recent study estimated that malaria may contribute to 3–5% of maternal anaemia, 8–14% of low birth weight (LBW) and 3–8% of infant mortality [10]. The harmful impact of malaria is most apparent in the first and second pregnancies of most pregnant women living in areas of relatively stable transmission [1].

Prevention of malaria in pregnancy is a major public health challenge and a priority for the Roll Back Malaria (RBM) Partnership. In high transmission areas including Nigeria, the RBM partnership recommends a three pronged approach for reducing the burden of malaria among pregnant women [11-13]. These are effective case management of malaria infection, use of insecticide treated nets (ITN) and intermittent preventive treatment (IPTp) in areas of stable transmission. In line with this recommendation, approach to prevention of malaria in pregnancy changed since the early 2000's, moving from a weekly or bimonthly chemoprophylaxis to intermittent preventive treatment (IPTp) and insecticide-treated bed nets (ITNs) [11]. Nigeria adopted the IPT strategy in year 2005 [13,14].

IPTp consists of administration of curative dose of an efficacious anti-malarial drug at least twice during the second and third trimesters of pregnancy during routinely scheduled antenatal clinic visits regardless of whether the woman is infected or not [9,15,16]. The drug is administered under supervision during antenatal care visits. Sulfadoxine-pyrimethamine (SP) is the drug currently recommended for the IPT strategy [11,14]. It has a good safety profile and remains a good option for IPTp in endemic areas in Africa [17,14]. The current National Malaria Treatment Guideline and Policy in Nigeria recommends SP as first line agent for IPTp and quinine for treatment of clinical malaria in all trimesters, Artemisinin based combination therapy (ACT) is considered safe second line agents in the second and third trimesters [12,13] and may be used in first trimester where there are no suitable alternatives [12]. In addition, ACT is recommended for treatment of uncomplicated malaria for the general populace following the development of resistance to chloroquine and SP.

IPTp with SP has been shown to reduce the risk of maternal anemia, placental parasitaemia and low birth weight [8,18,19]. In a study carried out in Ibadan, southwest Nigeria, IPTp-SP was found to be highly effective in preventing maternal and placental malaria among parturient women as well as in improving pregnancy outcomes such as delivery of bigger babies and lower prevalence of pre-term deliveries and maternal anaemia [9].

Antenatal Clinics are considered an important entry point to target the pregnant women [17,13] as 60–70% of women attend antenatal clinic at least once during any pregnancy in Nigeria [13,17]. SP as the drug of choice for IPTp (IPT-SP) is attractive because its single dose therapy lends itself for supervised administration in the antenatal clinic thus ensuring compliance [13].

Implementation of IPT strategy has been established in many health facilities in malaria endemic areas including Nigeria. However, it is estimated that less than five percent of pregnant women have access to effective malaria interventions; this is worse in the rural areas [20]. A survey carried out in four African countries showed that less than 20% of women use a prophylactic regimen close to the World Health Organization (WHO) recommendations [21]. As a result of this poor access, malaria remains one of the most important causes of maternal and childhood morbidity in sub-Saharan Africa [1,22].

To date only few studies have investigated factors affecting adherence to IPTp use [23]. The identified barriers to IPTp use are related to concerns about SP safety and poor understanding of the protocol among health care providers and the community [10]. In a study conducted in Tanzania, majority of respondents linked low compliance with IPTp to poor acceptance of SP because of perceived association of SP with side effects [17]. It was also reported that pregnant women throw away drugs after leaving the clinic. Other factors influencing compliance include late enrolment, periodic shortages of drugs and health workers underperformances [23].

Studies related to IPTp use during pregnancy in Nigeria are limited and none was found to have looked into compliance especially with the directly observed treatment (DOT) scheme. This study set out to assess IPTp use among pregnant women attending primary health centers for antenatal services in a rural area. Their knowledge, attitude towards IPTp use, compliance with IPTp and factors influencing IPTp use were determined. It was expected that findings from this study would provide valuable information to guide planning of programmes to improve the use of IPTp.

## Methods

### Study area

The study was conducted in Irepodun/Ifelodun local government area (LGA); one of the sixteen LGAs in Ekiti State, southwest Nigeria. It has a population of approximately 124,088 people who are predominantly Yoruba going by the provision of 1991 census [24], the major ethnic group in south western Nigeria. The major occupation of the inhabitants of the LGA is farming. Most of the women also engage in petty trading. Malaria is hyper-endemic in this LGA with perennial transmission. The health programmes of the local government is planned and managed by the Primary Health Care Department at the LGA headquarters. The local government area is rural and is divided into six health districts. There are thirteen health facilities; twelve primary health care centers which render antenatal services and one general hospital. Three primary health centers (PHC) rendering antenatal care are in the local government headquarter, Igede-Ekiti while one PHC each is located in nine other towns within the LGA. A Chief Nursing Officer heads each PHC and is assisted by staff nurses and community health workers. Antenatal care services are conducted on Mondays in the three health centers at the local government headquarters while the other health centers conduct clinics every Tuesday. Other activities at each PHC include distribution of free insecticide treated nets (ITNs) supplied by the Federal Ministry of Health and immunization.

### Study population

The study population comprised all consenting pregnant women attending antenatal care at all the primary health centers rendering antenatal services in the study LGA between July and August, 2007.

### Study design and sampling

A cross-sectional design was used. The sample size for the survey was calculated using estimate of reported IPTp use among pregnant women (16%) [17]. To detect IPTp use rate of 5% less or more than the reference rate at 95% confidence interval, the minimum number of pregnant women required for the study was 207. These were selected from the antenatal clinic attendees of the twelve primary health centers (PHCs) rendering antenatal services in the LGA using systematic sampling technique. The sample size was distributed among the PHCs based on proportionate to size allocation. The total ANC attendants for the previous one year in each of the facilities were used for the allocation. Using the estimate of the average clinic attendance of the month prior to the study, a sampling interval was determined for each PHC and systematic sampling was used to select the study subjects. The first pregnant woman to be interviewed was picked by balloting from the ANC appointment cards submitted to the record clerks.

### Data collection methods

Information was collected using interviewer-administered questionnaire, designed by the investigators and pre-tested prior to use [see Additional file 1]. The questionnaire was written in both English and Yoruba Languages and was administered by two trained local interviewers and one of the investigators (SA) in the language the respondents understood better. The questionnaire comprised of questions on socio-demographic characteristics, obstetric history, knowledge and attitude of pregnant women to malaria, antenatal clinic use, IPTp use and mothers' perception of the attitudes and activities of antenatal clinic staff.

### Ethical consideration

Ethical approval for this study was obtained from the Joint University of Ibadan/University College Hospital Institutional Ethics Review Committee. Verbal informed consent was obtained from each respondent before the interview.

### Data analysis

Data entry and analysis were performed using Statistical Package for Social Sciences (SPSS) version 13.0 SPSS Inc., Chicago, IL, USA. Data was summarized using frequency tables, graphs, means and standard deviations. Bivariate analysis was done with chi-square test or Fisher's exact test to compare proportions for categorical variables. Results were considered to be significant when the 2-sided value was < 0.05.

In order to assess the respondents' knowledge on IPTp, the responses to questions on the definition of IPT were rated as: 1 (very good) if respondents defined IPT as treatment for prevention of malaria during pregnancy, recognize SP as the drug of choice and the correct interval for IPT treatment. Respondents were rated 2 (average) if they knew that IPT was given to prevent malaria during pregnancy or that IPT is the use of SP during pregnancy and 3 (poor) if respondent could not define it correctly.

To further investigate the timeliness of ANC attendance, a categorization of 'early first attendance' was defined as a first visit to ANC at or before 4 months gestation, and those registering at fifth month till delivery were considered as "late first attendance" [25].

## Results

Two hundred and nine pregnant women participated in the study. The mean (SD) age of respondents was 25.1 (1.1) years with a range of 16 to 42 years and 64% in the age group 21–30 years. Many of the respondents 161(77.0%) were "Yoruba", half had secondary school education, majority, were Christians, 175(83.7%), married, 167(79.9%) and of multiple parity. Seventy three (34.9%), were traders and 53 (25.4%) were unemployed [Table 1].

**Table 1:** Socio-demographic characteristics of the respondents (N = 209)

**Variables**	**Frequency**	**Percentages**
**Age group (years)**		

16–20	42	20.1

21–25	63	30.1

26–30	71	34.0

31–35	26	12.4

> 35	7	3.3

**Level of Education**		

None	16	7.7

Primary	41	19.6

Secondary	105	50.2

Tertiary	47	22.5

**Marital Status**		

Single	39	18.7

Married	167	79.9

Divorced	2	1.0

Separated	1	0.5

**Occupation**		

Trading/farming	73	34.9

Unemployed	53	25.4

Civil servant	43	20.6

Artisan (e.g hairdresser)	40	19.1

**Monthly income**		

≤ N1000	4	1.9

N1000–N10000	26	12.4

N10000–N20000	16	7.7

> 20000	2	1.0

No sure income	161	77.0

**No of pregnancies**		

1–3	156	74.7

4–6	41	19.6

> 6	1	0.5

No responses	11	5.3

### Accessibility to PHC centres and time of registration at antenatal clinic

One hundred respondents (47.8%) walked to the clinic and 60 (58.8%) of these spent less than 10 minutes walking to the clinic. The mean (SD) length of time spent walking to the ANC was 10.00 (13.2) minutes. Fifty three (48.6%) of those that did not walk to the clinic spent less than10 minutes by transport to reach the clinic, mean (SD) of time spent by them was 15.80 minutes (13.2). Eighty six (41.1%) respondents spent between N50 and N100 (50 cents to one dollar – USD) for transportation to and from the clinic.

The mean (SD) gestational age of respondents at the first time they visited the clinic was 4.8 (2.15) months. Only 39 (18.7%) of the respondents first registered at the ANC in the first trimester, 117 (56.0%) did so in the second trimester and 44 (21.0%) in the third trimester. Nine (4.3%) of them could not remember when they first registered. When grouped into late and early first attendance, 96(46%) respondents registered early and 113 (54%) registered late.

### Attitude of pregnant women to taking drugs during pregnancy

Out of the 209 respondents, 68 (32.5%) mentioned they were afraid to take drugs in pregnancy, 73 (34.9%) said there were times they did not take drugs given to them in the clinic and 108 (51.7%) mentioned they will take drugs in clinic if allowed to use their own drinking cups. When those who had heard of IPTp were considered, 44 (40.4%) were afraid of taking drugs in pregnancy, 62 (56.9%) said they did not take drugs given to them in the clinic and 62 (56.9%) mentioned they will like to do so if allowed to use their own drinking cups in the clinic.

### Knowledge of IPTp

**Figure 1:**
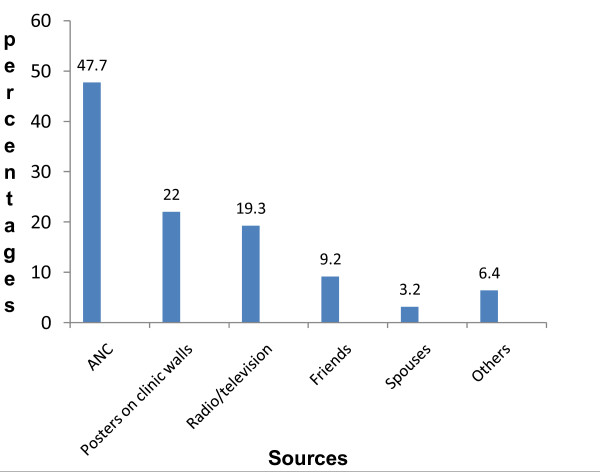
**The sources of information on intermittent preventive treatment of malaria in pregnancy (IPTp)**.

About half [109 (52.2%)] of the respondents, said they have heard about IPTp. The sources of information on IPTp are shown in Fig 1. Table 2 shows the rating of respondents' knowledge of IPTp, drug used, the dose and timing of IPTp use. Twenty six of the 109 (23.9%) who have heard about IPTp were able to give a good definition of IPTp and sixty-three (57.8%) said IPTp can be given to pregnant women. When asked when IPT drugs can be given during pregnancy, 67(61.5%) mentioned that it can be used between 4^th ^and 6^th ^months of pregnancy, 12 (11.0%) mentioned between 7^th ^and 9^th ^months and one mentioned 1^st ^to 2^nd ^months [Table 2]. About two thirds of those that have heard of IPTp (73/109; 67.0%) knew that SP is the recommended drug for IPTp. Using the different brand names of SP in the market, 13(17.8%) identified Fansidar^®^, 18(24.7%) identified Amalar^®^, 42(57.5%) identified Malareich^® ^which was the major brand given to them in the ANC clinic as drug used for IPTp. Forty nine (67.1%) of those who mentioned SP knew the correct dose of SP for IPTp.

**Table 2:** Respondents Knowledge of IPTp

**Variables**	**N = 109**	
	
	**Frequencies**	**Percentages**
**Definition of IPTp**		

1 = Very good	26	23.9

2 = Average	15	13.8

3 = Poor	68	62.4

*What drug is recommended for IPTp use		

Malareich	42	38.5

Amalar	18	16.5

Phensic	12	11.0

Chloroquine	15	13.8

Fansidar	13	11.9

*Who can be given IPTp		

Pregnant Woman	63	57.8

Infants	40	36.7

Aged people	20	18.3

Men	25	22.9

*How many tablets of IPTp is used as a dose		

3 tablets	51	46.8

2 tablets	26	23.9

5 tablets	3	2.8

1 tablet	3	2.8

*When is IPTp recommended to be used during pregnancy		

4–6 months	67	61.5

7–9 months	12	11.0

1–3 months	1	0.9

Don't Know	25	22.9

* Multiple responses

### IPT use in the index pregnancy

Fifty seven (27.3%) of the 209 respondents reported to have received at least one dose of IPTp during the index pregnancy and all were among those who have heard of IPTp (57/109; 52.3%). No significant difference in knowledge of IPTp was observed among those who had used IPT and those who did not use IPTp (χ^2 ^= 3.7, p = 0.16).

According to the description of use by respondents, 53 respondents mentioned that three tablets were dispensed to them, out of which 41 used the three tablets, giving compliance rate of (77.4%) [Table 3]. Twenty one of the 57 respondents (36.8%) who had received SP in current pregnancy used it in the ANC of which only three (3/21; 14.3%) were supervised by a health worker at the time of ingestion. Six out of the 21 (28.6%) that took the drug in the clinic used the cup provided by the clinic. Twenty two of the 36 women (61.1%) who did not take their drugs in the clinic would have liked to do so if allowed to bring their own drinking cups. Almost half (25/57; 43.9%) of those who had used IPTp during the index pregnancy expressed concern about possible adverse effect of SP on their pregnancies.

**Table 3:** Compliance with dose of IPTp drugs by respondents

	**Tablets of SP used**		
**Tablets of SP given**	**Incomplete** n (%)	**Complete** n (%)	**Total** n (%)

Incorrect (<3 Tablets)	4 (1.0)	0 (0)	4 (7.0)

Correct (3 Tablets)	12 (21.1)	41 (71.9)	53 (93.0)

**Total**	**16(28.1)**	**41 (71.9)**	**57(100.0)**

P = 0.001, df = 1, X^2 ^= 11.024 (significant)

Thirty six (63.2%) of those who had received IPTp booked for antenatal care in the 2nd trimester, 13 (22.8%) in the 1st trimester and 4 (7.0%) in the 3rd trimester. The remaining four could not say when they first registered for antenatal care. Only 9 (15.8%) had received the second dose of IPTp by the time of interview. Pregnant women who registered in the 1st and 2nd trimester (p = 0.02; χ^2 ^= 10.1; df = 3) and those who were able to define IPTp correctly (p < 0.001; χ^2 ^= 23.4; df = 2) were significantly more likely to receive IPTp. The number of women who received IPTp increased as the number of pregnancies increased up to the third pregnancy and decreased thereafter (p = 0.01; χ^2 ^= 17.9; df = 7). Majority of the women 116 (77.0%) did not have a sure source of monthly income. However, 29 out of 48 who volunteered the amount they earned monthly earned less than N10000 monthly (< $100 USD) and these group of women were more likely not to use IPTp (p < 0.001; df = 3; χ^2 ^= 2). There was no significant relationship between gestational age, age of respondents, and length of time (minutes) spent trekking to the clinic and IPTp use (p > 0.05).

## Discussion

Overall, respondents' knowledge, attitude and practice of IPTp were poor in this study. Majority did not know sulfadoxine-pyrimethamine (SP) as the drug recommended for IPTp and were not aware that IPTp could be given to pregnant women. This is unlike findings in a study in Tanzania, which reported that pregnant women were generally aware of SP as the drug recommended for IPTp [17]. Half of the respondents in this study had heard about IPTp but only a third of these could explain what IPTp is. However, many of these demonstrated good knowledge of the drug used, who could be given and when it is given during pregnancy. In addition many of those who knew SP as drug for IPTp also knew the correct dose. These findings underscore the need to create more awareness and improve specific knowledge on IPTp among pregnant women and those of child bearing age.

The WHO expects 80% of all pregnant women living in areas of high transmission to receive IPTp during pregnancy by 2010 [26]. However, the coverage of the intervention is still low. In Kenya, one of the first countries to implement IPTp, the national coverage with two doses of SP was only 4% five years after IPTp implementation [27]. Malawi recorded the highest coverage of 60% close to achieving the 2000 Abuja target by 2005 [28]. In this study only 27.3% had received a dose of SP during the index pregnancy. The probable reason for the low uptake is the low level of awareness and poor knowledge of IPTp by the pregnant women. This is supported by the fact that those who were able to define IPTp correctly in this study were more likely to have received IPTp at least once.

Worst still regarding IPTp use is the poor adherence to the recommendations for use. Compliance with the recommendation that IPTp drug should be given under supervision in the clinic was very low in this study. Only about a third of the respondents who received IPTp used SP in the clinic and only three of them were supervised by a health worker at the time of ingestion, giving a directly observed therapy (DOT) compliance rate of 14.3%. This low use of IPTp and poor adherence may not be peculiar to southwest Nigeria alone and the findings in this study may be a proxy to the practice of IPTp in PHC centres in other parts of the country. Previous related studies in eastern and northern Nigeria reported low knowledge of malaria in pregnancy and management practice as well as poor maternal health care [22,29].

The reason for poor adherence could be patient or health worker related. In two cross-sectional studies funded by the World Health Organization in Muheza district and Mpwapwa district in Central Tanzania cited by Mubyazi et al, 2005, low compliance with the use of SP was partly attributed to health care providers' and users' fear of side effects of SP and their inadequate knowledge of the correct dose. Similarly, findings in another study conducted in Tanzania reported 74% of respondents were said to have believed that antimalarial drug when taken during pregnancy could be harmful to the pregnant women and the unborn children [17]. Mbonye et al. (2006) reported that pregnant women in Uganda believed that SP is strong and weakens pregnant women, causes abortions and fetal abnormalities [30]. In this study, respondents expressed concern that the drug used for IPTp may cause complications during pregnancy.

One other user related reason for low compliance was the unfavorable disposition of the respondents to the use of cups provided in the clinic. This made them to opt for taking SP at home. Allowing pregnant women to take the IPTp drug unsupervised be it at the clinic or at home makes compliance uncertain and undermines the essence of IPTp. This practice is probably due to concern about the quality of water and hygienic use of cups provided in the clinics as shown in related past studies. For example, in a study in Tanzania, one of the district medical officers interviewed opined that the DOT scheme would be effective if the problem with the shortage of clean water and sharing of cups in the clinic at peripheral health facilities was solved. This was alluded to in this study whereby respondents said they would be willing to take the IPTp medication in the clinic if they were allowed to bring cups and water from home. It is also possible that health workers are not well informed about the need to give IPTp medication supervised or there is laxity in enforcing the regulation.

Regular availability of SP in the health facility is also critical to improving the use of IPTp and implementation of DOT scheme [17]. Sulfadoxine-pyrimethamine for IPTp in public hospitals in Nigeria was supplied by the Federal Ministry of Health up to the time of this study. However, periodic shortages of supply were reported by respondents.

In this study, the health facilities were found to be easily accessible to the pregnant women, as many of them could get to the clinic within ten minutes of walking or by public transport. In addition transport fare was also minimal. The provision of functional PHCs in every health district by the LGA is commendable as the easy accessibility is expected to impact positively on the health of the community and be an encouragement for utilization of primary health centre for antenatal care. However, only half of respondents in this study were early attendants suggesting there may be other reasons for the pregnant women not attending clinic early. A study that compared community based delivery system for IPTp with IPTp at health units in area of high transmission in Uganda showed that the community-based approaches increased access and adherence to IPT with an effect on anemia, severe anemia, parasitemia and low birth weight [31]. This could be another option that could be adopted in addition to improving level of awareness and knowledge to increase the coverage of IPTp and adherence.

In this study, the mean (SD) gestational age of respondents as at the first time they visited the clinic was 4.8 (2.15) months. Only about a fifth of the respondents first attended ANC in the first trimester of gestation, half did so in the second trimester while another fifth registered in the third trimester. This is similar to a study carried out in northern Nigeria whereby 63% registered for ANC in their 2^nd ^trimester [30]. When proportion of early first attendance was considered, the finding was similar to that of a study in Tanzania which found about half of the women to have first attended ANC during or before the fourth month of gestation [25]. This late registration at ANC has implications for uptake of IPTp. With the late registration it is unlikely that pregnant women will take the recommended two doses of SP. Late first ANC attendance has been identified as an important factor contributing to incomplete IPT use [27]. The decision that IPTp be administered through ANC was informed by the expectation that pregnant women will attend clinic frequently enough to allow for two doses of SP for IPTp [23]. Although ANC attendance is high in most countries with IPTp policy (median, 2.0–4.8 ANC visits per woman) [23], it has not been sufficient to ensure a high IPTp coverage.

This study was carried out in only one of the 774 LGAs in Nigeria hence cannot be generalizable. However, it has provided useful information that can guide policy and highlighted the need to carry out similar studies in the other geopolitical zones in the country. This is a hospital-based study. A community-based study will better capture knowledge of women who have not been exposed to health facilities especially antenatal clinic.

## Conclusion

The IPTp strategy as currently implemented in this study area falls short of ensuring that Nigeria achieves the target of 80% coverage by 2010. These findings highlight issues to be addressed in order to achieve the target. These include (i) low level of awareness on IPTp (ii) low uptake among pregnant women and (iii) poor adherence to the DOT scheme. Highlighted also in this study are some barriers to IPTp use, which include poor knowledge by respondents, late enrolment for antenatal care, non-acceptability of the use of drinking cups provided in clinic for DOT scheme and periodic shortages of IPTp drugs in the clinic.

The plausible interventions to address the gaps and deficiencies include developing a health promotion package to explain the benefits of SP as IPTp agent and to counteract the wrong perception that SP could harm both mother and child. In addition health workers should be provided continuing education and training to improve their knowledge about malaria during pregnancy and in particular IPTp strategy and DOT scheme. Activities of health workers manning the antenatal clinics should also be supervised. Special information, communication and education package to create awareness of the general public on the use and safety of SP in pregnancy with efforts to target special groups like pregnant women and adolescents should be provided.

## Competing interests

The authors declare that they have no competing interests.

## Authors' contributions

SA and IA conceived the study; SA, IA and CF participated in research design. SA supervised data collection from the field and data analysis. SA, IA and CF contributed to data interpretation and writing of the draft. IA was the overall supervisor of the project and revised the draft extensively. All authors read and approved the final manuscript.

## Pre-publication history

The pre-publication history for this paper can be accessed here:



## Supplementary Material

Additional file 1 **Questionnaire sent with manuscript-Intermittent preventive therapy (ipt) use among pregnant women attending antenatal clinics in primary health centers of Irepodun/Ifelodun local government area, Ekiti state**. This is the questionnaire that was used to collect information from the respondents. Click here for file
