# Supplementary material for: Knowledge and utilization of intermittent preventive treatment for malaria among pregnant women attending antenatal clinics in primary health care centers in rural southwest, Nigeria: a cross-sectional study
Source: BMC Pregnancy Childbirth. 2009 Jul 9;9:28. doi: 10.1186/1471-2393-9-28 (PMC2719593; doi:10.1186/1471-2393-9-28)
Supplement: Additional file 1 — Questionnaire sent with manuscript-Intermittent preventive therapy (ipt) use among pregnant women attending antenatal clinics in primary health centers of Irepodun/Ifelodun local government area, Ekiti state. This is the questionnaire that was used to collect information from the respondents. [file 1471-2393-9-28-S1.doc]

APPENDIX 1

INTERMITTENT PREVENTIVE THERAPY (IPT) USE AMONG PREGNANT WOMEN ATTENDING ANTENATAL CLINICS IN PRIMARY HEALTH CENTERS OF IREPODUN/ IFELODUN LOCAL GOVERNMENT AREA, EKITI STATE.

Dear Respondents,

This is a health survey questionnaire to study Intermittent Preventive Therapy (IPT) use among pregnant women. All information would be treated confidentially. Refusal to participate will not in any way affect the quality of care that will be provided.

Please kindly ensure that you answer all questions truthfully.

Thanks for your cooperation.

Date: ………………………………… ANC Clinic: ……………………………

Serial no: ………………………………

SECTION A (Relevant Socio-Demography Information).

1. Age………………………………

2. Ethnic group. (1) Yoruba (2) Ibo (3) Hausa (4) Others (Please Specify)

3. Level of Education.

1. Didn’t go to school

2. Primary only

3. Secondary only

4. Higher Institution

5. Others e.g. Koranic School.

4. Religion (1) Christianity (2) Islam (3) Traditional (4) Others (Please Specify)

5. Marital Status (1) Single (2) Married (3) Divorced (4) Separated

6. Occupation? ………………….

7. How much do you earn per month? ………………………….

( Accessibility to ANC)

8. How many minutes will it take you to get to the ANC Clinic from your house by taking a taxi or by walking? ………………………..specify answer

9. Cost of transportation to and from ANC Clinic? ………………….

SECTION B (Obstetric History)

10. What is your present gestational age? ……………………………

11. How many deliveries have you ever had? . ...................................

12. How many pregnancies have you ever had? ……………………….

SECTION C (Knowledge and Attitude of pregnant women towards Malaria in pregnancy)

13. What is Malaria? …………………………………………….

…………………………………………………………………….

…………………………………………………………………..

14. How is malaria transmitted ………………………………………

(a) mosquito bites (b) house flies (c) termites (d) cockroaches (e) others, please specify ……………………………………….

15. The following encourages malaria transmission? …………………………… (a) Dirty environment 1. Yes 2. No 3. Don’t know

(b) Lakes, pits, dams, around surroundings 1. Yes 2. No 3. Don’t know

(c) Clean houses 1. Yes 2. No 3. Don’t know

(d) Ill ventilated and Ill-lighted houses 1. Yes 2. No 3. Don’t know

16. Malaria affects all age groups? 1. Yes 2. No 3. Don’t know

17. Pregnant women don’t have Malaria? 1. Yes 2. No 3. Don’t know

18. Effects of malaria in pregnancy include

(a) Tuberculosis 1. Yes 2. No 3. Don’t know

(b) HIV 1. Yes 2. No 3. Don’t know

(a) Maternal anaemia 1. Yes 2. No 3. Don’t know

(b) Placental parasiteamia 1.Yes 2. No 3. Don’t know

(c) Still birth 1. Yes 2. No 3. Don’t know

(d) Maternal death 1. Yes 2. No 3. Don’t know

(e) Low birth weight of baby 1. Yes 2. No 3. Don’t know

(f) Abortion 1. Yes 2. No 3. Don’t know

SECTION D (History of malaria during pregnancy)

19. Have you ever had malaria during pregnancy? 1. Yes 2. No

20. During the attack, what was your first treatment……….

(a) Did you go to the Hospital? 1.Yes 2. No

(b) Did you use herbal medicine? 1. Yes 2. No

(c) Did you treat yourself at home? 1. Yes 2. No

(d) Did you use any drug bought in the chemist? 1. Yes 2. No

(e) Any other treatment, please specify ………………………………….

(f) Were you cured? 1. Yes 2. No

21. What was your second treatment? ………………………..

59. Any other recommendation for the prevention of malaria during pregnancy……………………………………………………….

…………………………………………………………………

SECTION E (Antenatal use by pregnant women and attitude of antenatal staffs)

22. Is this your first time of coming to ANC Clinic during this pregnancy? 1. Yes 2. No

23. If No, What age was your pregnancy when you started coming? …...

24. Do you always keep your appointments? 1. Yes 2. No

If No, why? ...................................................

25. Is transportation fare a barrier to keeping your appointments? . 1. Yes 2. No

26. Do health Nurses give talks on malaria? ......................................

27. What do they educate you about? ...............................................

28. How would you rate the attitude of the ANC staffs in your Clinic?

(1) caring 1. Yes 2. No 3. Average

(2) always polite 1. Yes 2. No 3. Average

(3) takes good care of us 1. Yes 2. No 3. Average

(4) always shout at 1. Yes 2. No 3. Average

(5) Any other, please specify …………………………………

SECTION F (Practice of IPTp and ITNs use )

29. Do you know Insecticide treated Nets (ITNs)? 1. Yes 2. No

If yes, Insecticide Treated Nets (ITNs) is

(a) Used for treating Malaria 1. Yes 2. No

(b) Used for preventing mosquito bites 1. Yes 2. No

(c) Used for preventing Malaria 1. Yes 2. No

30. What is the difference between ITN and other mosquito nets? ……..

……………………………………………………………………..

31. Do you use Insecticide Treated Nets 1. Yes 2. No

If No, why ……………………………………………….

32. Do they give insecticide treated Nets in this centre? 1. Yes 2. No

33. Where did you get your ITN? ………………………………………

34. How much did you get your ITN? …………………………………

35. Have you heard about Intermittent Preventive Therapy IPT? 1. Yes 2. No

36. From where did you hear about IPT? ……………………….

(a) Friends (b) Husband (c) Radio or Television (d) Hospital posters (e) ANC Clinic (f) others, please specify…………………….

37. Do you know Intermittent preventive therapy (IPT)? 1. Yes 2. No

If yes, what is IPT? ………………

38. What Drug is recommended for IPT use?

(a) Chloroquine 1. Yes 2. No 3. Don’t know

(b) Fansidar 1. Yes 2. No 3. Don’t know

(c) Phensic 1. Yes 2. No 3. Don’t know

(d) Amalar 1. Yes 2. No 3. Don’t know

(e) Malareich 1. Yes 2. No 3. Don’t know

39. Intermittent Preventive Therapy can be given to?

1. Men 1. Yes 2. No 3. Don’t know

2. Pregnant Women 1. Yes 2. No 3. Don’t know

3. Aged People 1. Yes 2. No 3. Don’t know

4. Infant 1. Yes 2. No 3. Don’t know

40. How many tablets of IPT drug is being used at once as a dose?

1. 1 tablet 2. 2 tablets 3. 3 tablets 4. 4 tablets 5. 5 tablets

41. When is IPT Doses recommended to be used during pregnancy?

1. 1st- 3rd months 2. 4th- 6th months 3. 7th- 9th months 4. 2nd- 4th months

42. Who are those not suppose to use IPT? …………………….

43. Since your coming to this ANC center have you receive IPT drug? 1. Yes 2. No 3. Don’t know

44. How many tablets were you being given? ………………………

45. How many did you use? …………………………

46. Where did you use it? 1. Home 2.In the Clinic 3.Outside the clinic

47. How much did you pay for the drug? ………………………

48. When you used it, were you being Supervised? 1. Yes 2. No

If Yes, who supervised you? .................................................

49. Did you use the clinic cups provided for use? 1. Yes 2. No

50. Do you like taking the drugs in the clinic? 1. Yes 2. No

51. Is there any time you didn’t take the drugs given to you in the clinic?

1. Yes 2. No

52. Is there any time you were afraid of any complication during pregnancy and so didn’t use the drug? 1.Yes 2. No

53. Is there any time you used IPT during pregnancy and still had malaria? 1. Yes 2. No

54. If Yes, which of your pregnancies? ..........................................................

55. How many dose did you take?

1.1 dose 2. 2 doses 3. 3 doses 4. 4 dose 5. 5 doses

56. When did you take your first dose? ………………….

57. For each dose, how many tablets did you use? …………………….

58. After using IPT, was there any side effect? 1. Yes 2. No

If Yes, Please specify ………………………………..

59. Any other recommendation for the prevention of malaria during pregnancy……………………………………………………….

…………………………………………………………………

60. What would you suggest to improve IPT use in the Clinics………….

………………………………………………………………………
